# Myths, facts and controversies in the diagnosis and management of anaphylaxis

**DOI:** 10.1136/archdischild-2018-314867

**Published:** 2018-06-16

**Authors:** Katherine Anagnostou, Paul J Turner

**Affiliations:** 1 Department of Pediatrics, Section of Immunology, Allergy and Rheumatology, Texas Children’s Hospital, Houston, Texas, USA; 2 Pediatrics, Section of Immunology, Allergy and Rheumatology, Baylor college of Medicine, Houston, TX, USA; 3 Section of Paediatrics, Imperial College London, London, UK

**Keywords:** allergy, anaphylaxis, food allergy, vaccines, adrenaline

## Abstract

Anaphylaxis is a serious systemic allergic reaction that is rapid in onset and may cause death. Despite numerous national and international guidelines and consensus statements, common misconceptions still persist in terms of diagnosis and appropriate management, both among healthcare professionals and patient/carers. We address some of these misconceptions and highlight the optimal approach for patients who experience potentially life-threatening allergic reactions.

## Introduction

Anaphylaxis is a serious systemic allergic reaction that is rapid in onset and may cause death.^1^ Recent data suggest that the incidence is increasing, particularly to food.^2–4^ The lifetime prevalence of anaphylaxis is estimated to be between 0.5% and 2%.^5^ Despite numerous national and international guidelines, misconceptions continue to persist among both healthcare professionals and patients/carers, which result in under-recognition and suboptimal management of this medical emergency. In this review, we address some of these misconceptions and highlight areas of best practice.

### Myth 1: ‘Anaphylaxis often results in death’

Anaphylaxis can be life-threatening, but in reality the majority of reactions do not result in severe outcomes.^6 7^ Many reactions are not treated appropriately (discussed below), yet fatal anaphylaxis is (fortunately) a rare event, with a case fatality rate under 0.001%.^8^ Severe anaphylaxis, however, is unpredictable, and severe reactions may mimic more mild anaphylaxis reactions in the first instance.^9^ Delay in appropriate treatment almost certainly contributes to fatalities.^10^ Therefore, *it is critical that all anaphylaxis reactions are treated as a medical emergency*.

While hospitalisations in the UK and elsewhere due to anaphylaxis have increased over the last two decades, there has been no increase in fatalities.^2 11–14^ For the food-allergic individual, the incidence of fatal anaphylaxis is 1.81 per million person years—less than death due to accidental causes or murder.^7 15^ Nonetheless, this needs to be interpreted appropriately: allergic individuals (and their parents) perceive risk very differently: a ‘one in a million’ risk may be acceptable in terms of public health but with respect to their own child, parents will consider their child to be the ‘one in a million’ who will die from anaphylaxis.^16^ Indeed, the adverse impact of a diagnosis of food allergy on health-related quality of life is greater than that seen in diabetes and other chronic diseases. These data are perhaps best framed in the context of safety-netting: just as we manage everyday risks (such as driving, with safety standards on cars, airbags and crumple zones, adhering to a highway code), can we help our patients and their families take a similar approach to the food allergy, with safety-netting allowing affected individuals to lead as normal a life as possible?

## Diagnosis of anaphylaxis

Anaphylaxis has been defined as a systemic or multiorgan allergic reaction; however, not all systemic reactions are anaphylaxis. For example, many reactions have only cutaneous manifestations (eg, generalised urticaria)—clearly a systemic phenomenon, but (in the absence of other symptoms) not anaphylaxis according to most guidelines. In practice, anaphylaxis in the UK (and also Australia) is *characterised by the presence of ‘Airway/Breathing/Circulation’ (respiratory or cardiovascular) symptoms* as part of an allergic reaction. Skin or mucosal changes alone are not a sign of an anaphylactic reaction.

There are two areas of potential controversy: the most common criteria to diagnose anaphylaxis are those developed by the National Institute of Allergy and Infectious Diseases (NIAID) and subsequently adopted by the World Allergy Organisation (box 1),^1^ which were designed to capture 95% of cases. However:

1. According to criterion 2, skin and gut symptoms together constitute anaphylaxis. However, the prevailing consensus in the UK (and Australia) with respect to food-induced reactions is that skin and gut symptoms, in the absence of respiratory or cardiovascular symptoms, are *not* anaphylaxis.^17^ For food, gastrointestinal symptoms are caused by the presence of *local* allergen in the gut rather than a systemic reaction. This is in contrast to venom-induced reactions, where the presence of gastrointestinal symptoms (eg, vomiting) would constitute anaphylaxis (as the gut is remote from the site of allergen exposure).^17^ There is also no consensus as to what constitutes *persistent* gut symptoms. This distinction is important, as many food-induced reactions are classified as anaphylaxis in the USA (and therefore should be treated with epinephrine), but not in the UK and Australia, something important to consider when making comparisons to US data.
2. Fatal (and in our experience, near-fatal) anaphylaxis reactions often present as acute bronchoconstriction without any other symptoms being present (which often leads to uncertainty as to whether some fatalities are due to anaphylaxis or severe asthma).^18 19^ Such reactions, according to the NIAID criteria, do not constitute anaphylaxis.

Box 1 **Clinical criteria for the diagnosis of anaphylaxis^1^**

Anaphylaxis is highly likely when any one of the following three criteria is fulfilled:

1. Acute onset of an illness (minutes to several hours) with involvement of the skin, mucosal tissue or both (eg, generalised urticaria, itching or flushing, swollen lips–tongue–uvula)
  And at least one of the following:
  - Respiratory compromise (eg, dyspnoea, wheeze-bronchospasm, stridor, reduced peak flow, hypoxaemia)
  - Reduced blood pressure or associated symptoms of end-organ dysfunction (eg, hypotonia (collapse), syncope, incontinence) OR
2. Two or more of the following that occur rapidly after exposure to a likely *allergen* for that patient (minutes to several hours):
  - Involvement of the skin–mucosal tissue
  - Respiratory compromise
  - Reduced blood pressure or associated symptoms
  - Persistent gastrointestinal symptoms (eg, crampy abdominal pain, vomiting) OR
3. Reduced blood pressure after exposure to known *allergen* for that patient (minutes to several hours)

Respiratory symptoms are far more common than cardiovascular symptoms in food-induced anaphylaxis, especially in those with asthma.^20^ In a retrospective study from Sweden, children with asthma presenting with anaphylaxis were more likely to have lower airway symptoms and wheeze than children without an underlying diagnosis of asthma (OR 2.7).^21^

### Myth 2: ‘There are no hives so it can’t be anaphylaxis’

Cutaneous symptoms (most commonly urticaria or ‘hives’) are absent in around 10% of anaphylaxis reactions and where present may be delayed in onset.^8^ This is consistent with a case series of six paediatric fatalities due to food anaphylaxis, where only one child had evidence of skin involvement: the lack of skin signs may have delayed diagnosis and appropriate treatment with epinephrine, contributing to the fatal outcome.^22^ The Australasian Society of Clinical Immunology and Allergy (ASCIA) recently issued new guidelines,^17^ which define anaphylaxis as:

- Any acute onset illness with typical skin features (urticarial rash or erythema/flushing, and/or angio-oedema), PLUS involvement of respiratory and/or cardiovascular and/or persistent severe gastrointestinal symptoms; or
- Any acute onset of hypotension or bronchospasm or upper airway obstruction where anaphylaxis is considered possible, even if typical skin features are not present.

These criteria better reflect increasing recognition that cutaneous manifestations are often absent or appear late in near-fatal and fatal anaphylaxis.

**Figure 1:**
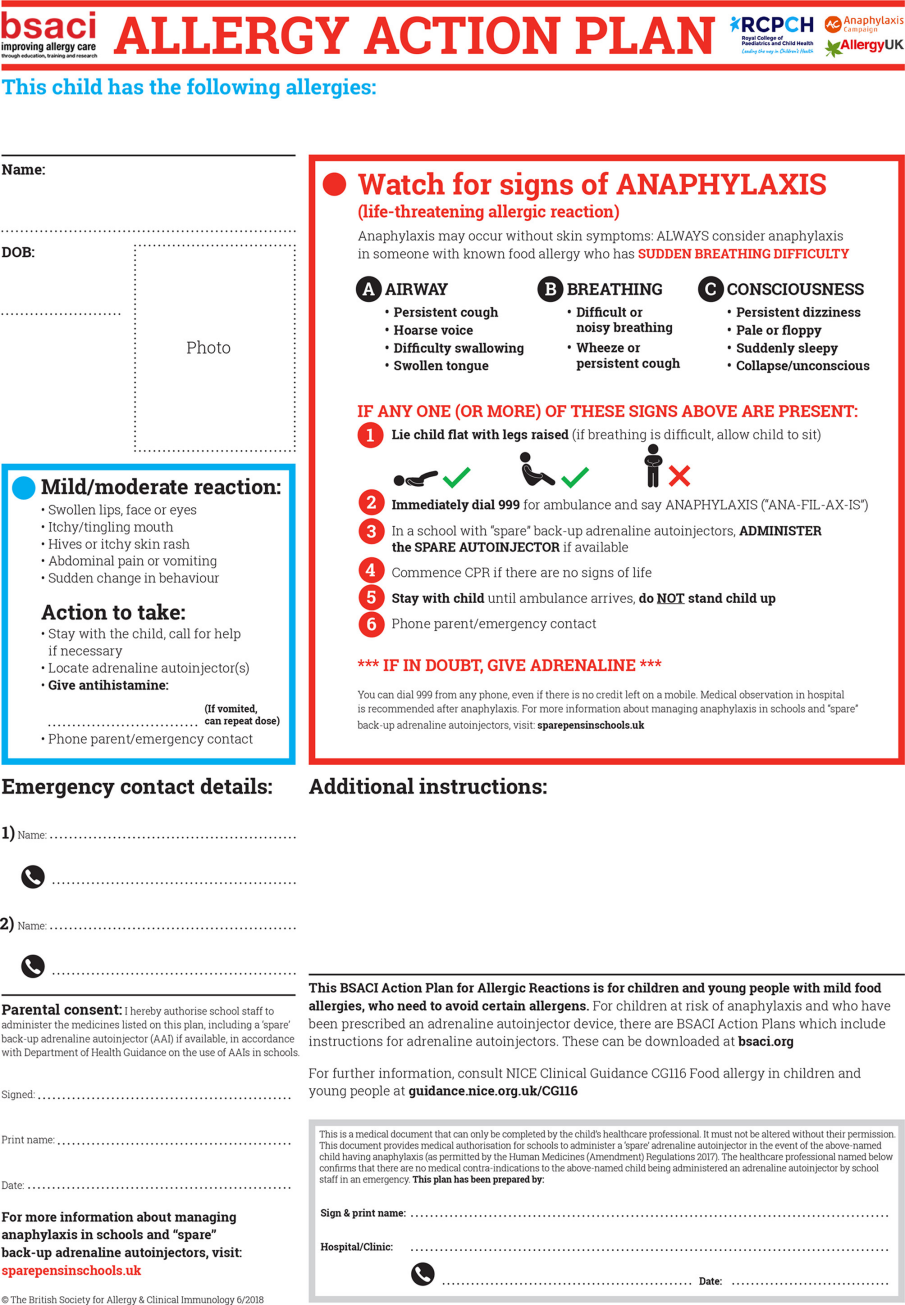
Allergy Action Plan from the British Society for Allergy and Clinical Immunology/Royal College of Paediatrics and Child Health (available at www.sparepensinschools.uk or http://www.bsaci.org/about/pag-allergy-action-plans-for-children).

*The safe management of anaphylaxis depends on early recognition and treatment with intramuscular epinephrine.* The British Society for Allergy and Clinical Immunology (BSACI), in conjunction with the Royal College of Paediatrics and Child Health, has recently updated its Allergy Management Plans for children (figure 1), highlighting the potential for skin symptoms to be absent in anaphylaxis.

### Myth 3: ‘No trigger for the reaction is identified, therefore it is not anaphylaxis’

Anaphylaxis is a *clinical* diagnosis. The most common trigger in young people is food: symptoms typically begin within 15–30 min of exposure and progress rapidly.^23^ Other triggers, such as medication or insect stings, are far less common in children.^8 24^ In around 20% of cases, no trigger is identified; this is known as idiopathic anaphylaxis. Many such reactions will be due to undisclosed or ‘hidden’ food allergens. Identifying the culprit allergen can be challenging and referral to an allergy specialist is advised: a thorough review of the circumstances surrounding the reaction including a detailed dietary history supported by ingredients lists is likely to be required. Of note, if a child is consuming a food regularly without problem, it is unlikely to be the cause. This might seem obvious, but dietary manipulation along these lines are often recommended by non-specialists for idiopathic, non-anaphylaxis reactions presenting with only skin symptoms: such episodes are generally due to immune activation (often viral-triggered) rather than allergen exposure.

*The most common food trigger for fatal anaphylaxis in children in the UK is milk,* followed by peanut and tree nuts.^2^ While there is broad public recognition of the risks posed by nuts, cow’s milk allergy is often perceived as being less severe. However, milk allergy persisting into school age is often associated with other coexisting atopies (such as asthma) and more severe reactions, particularly in the 30%–40% of milk-allergic children who are unable to tolerate milk in well-baked foods (such as biscuits or cakes).^9^ Such exposure often results in delayed reactions which mimic asthma; under such circumstances, it may not be obvious that the child has been exposed to milk. Therefore, *always consider anaphylaxis in someone with a known food allergy who has sudden breathing difficulty.*

Laboratory tests (such as mast cell tryptase, MCT) may support a diagnosis of anaphylaxis, but these are not specific for anaphylaxis, nor are results available quick enough to impact on acute management.^25^ Measuring MCT may be helpful where the cause of the reaction is unclear: a serum sample should be collected within 15–180 min of symptom onset, with a further convalescent sample at least 6 hours later.^25^ However, MCT is often not raised in food-induced reactions, even in the most severe and fatal reactions.^26^ In a Canadian study, only 19.2% of children presenting with anaphylaxis had elevated MCT; even with severe reactions (cyanosis, hypoxia, respiratory arrest, hypotension, loss of consciousness), MCT was only raised in 50% of cases.^27^ A negative MCT does not, therefore, rule out anaphylaxis.

## Acute management

*Epinephrine is the first-line treatment for anaphylaxis* according to all guidelines.^10^ It has both α-sympathomimetic and β-sympathomimetic actions, causing peripheral vasoconstriction, increased cardiac output and bronchodilation; importantly, it is the only drug that inhibits the further release of inflammatory mediators from mast cells and basophils.

### Myth 4: ‘Epinephrine is dangerous’

Epinephrine given by intramuscular injection into the outer mid-thigh is very safe and starts to work within minutes. Epinephrine can either be injected using a needle–syringe (using 1:1000 epinephrine, which results in a lower volume, less painful injection than if using 1:10 000) or by autoinjector device (eg, Emerade, EpiPen, Jext). Where an autoinjector is used, note that both EpiPen and Jext are only available in 150 µg and 300 µg doses, which means that the 300 µg is effectively an underdose in someone over 30 kg (this may explain why some patients require a second epinephrine dose). Younger children should be transitioned to a 300 µg dose when their body weight is >25 kg, and some centres advocate doing so from 20 kg. Around 10%–20% of patients report transient effects including pallor, anxiety, palpitations, dizziness and headache (although these symptoms may also be due to the reaction and/or the patient’s own endogenous epinephrine production).

**Figure 2:**
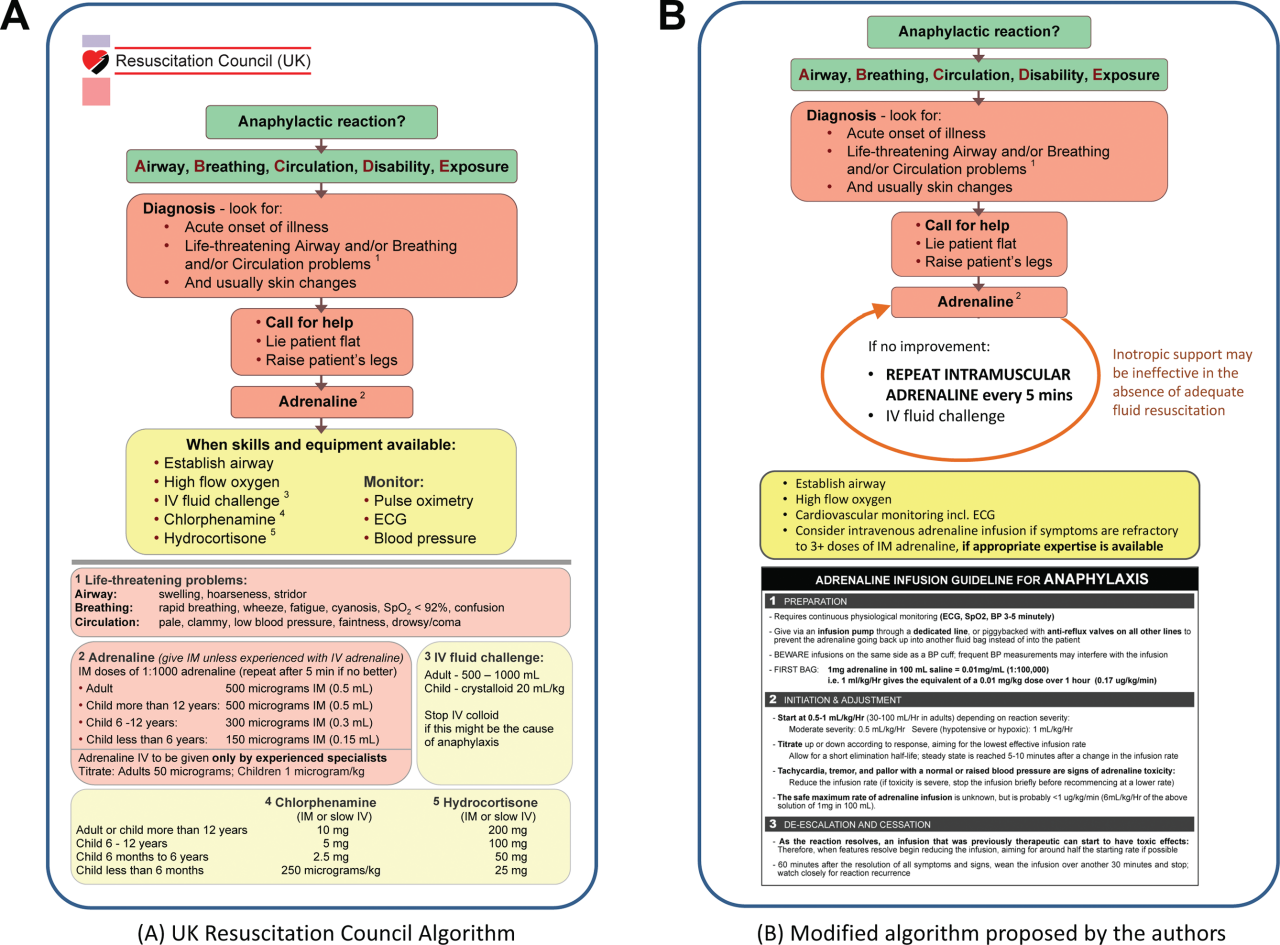
Acute management of anaphylaxis. (A) Current UK Resuscitation Council algorithm. (B) Suggested amended algorithm by the authors, which emphasises the need for further doses of intramuscular epinephrine in the event of ongoing anaphylaxis symptoms and incorporates a low-dose epinephrine infusion protocol used widely in Australia and Spain (with permission, from Brown SG, *Emerg Med Australas*. 2006;18:155–69).

Epinephrine is underused in the treatment of anaphylaxis, both prehospital and in emergency departments.^6 10 21 23 28^ Further intramuscular doses of epinephrine should be administered in the event of persisting respiratory or cardiovascular symptoms. Epinephrine can and should be repeated after 5 min; the administration of other medication such as antihistamines or steroids must not cause delay or distraction, as these are not first-line (or even second-line) treatments for anaphylaxis^24^ (figure 2A). An alternative summary of anaphylaxis treatment, consistent with national and international guidelines, is shown in figure 2B.

### Myth 5: ‘Antihistamines can be used to treat anaphylaxis initially; epinephrine is only needed if symptoms worsen’

Histamine is only one of many inflammatory mediators released during anaphylaxis. Oral antihistamines take around 30 min for onset of effect; intravenous chlorphenamine has a faster onset, but can cause hypotension. Antihistamines are not effective against anaphylaxis: their prophylactic use during controlled immunotherapy does not prevent anaphylaxis, and any apparent response during acute management of reactions is most likely due to the patient’s own endogenous epinephrine.^29^ Antihistamines have now been relegated to third-line therapy in international guidelines; their use is limited to the relief of cutaneous symptoms and should never delay the administration of epinephrine or fluid resuscitation during patient stabilisation.^10^

### Myth 6: ‘Corticosteroids prevent delayed or biphasic reactions in anaphylaxis’

Historically, corticosteroids have been used to prevent protracted and biphasic reactions (the latter defined as a recurrence of symptoms within 72 hours of initial anaphylaxis, without re-exposure to the trigger). However, this has never been tested in a randomised clinical trial; more recent evidence has cast doubt over their efficacy.^30^ A recent systematic review and meta-analysis included 27 studies with 4114 anaphylaxis cases, of whom 192 (4.7%) had biphasic reactions.^31^ Steroid administration did not affect the likelihood of a late phase reaction (OR 1.52, 95% CI 0.96 to 2.43). In fact, there was a non-significant trend towards increased risk, although this is probably because steroid use was more common with severe reactions. Biphasic reactions were more common where hypotension was present at initial reaction (OR 2.18, 95% CI 1.14 to 4.15), but this is unusual in food-induced anaphylaxis. The median time to onset of biphasic symptoms was 11 (range 0.2–72) hours, that is, 50% of reactions occurred >11 hours *after* initial reaction. This is relevant because current guidance from the National Institute for Health and Care Excellence recommends patients over 16 years are observed for 6–12 hours after anaphylaxis (children under 16 should be admitted).^32^ In reality, it is generally accepted that prolonged observation may not be required following a straightforward reaction in someone who already has a comprehensive management plan and rescue medication (including epinephrine autoinjectors) in place.

## Managing children at risk of anaphylaxis

Although research in ongoing into potential treatments for food allergy, the mainstay of management remains dietary avoidance and provision of a management plan/rescue medication in the event of accidental reactions.

### Myth 7: ‘Only children who have had anaphylaxis need an epinephrine autoinjector’

**Figure 3:**
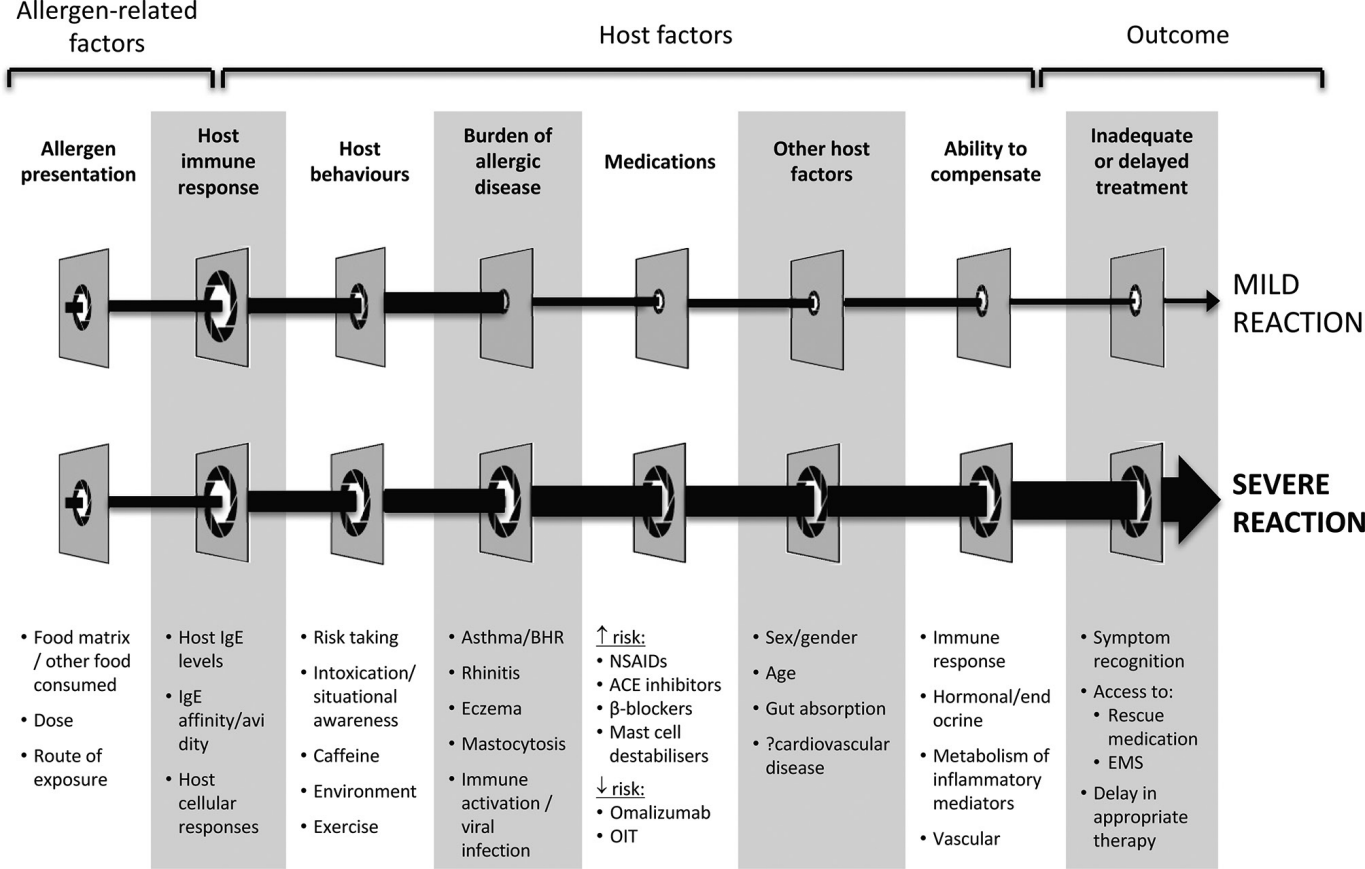
Risk factors for severe reactions. Reproduced with permission from Dubois *et al.* 34 BHR, bronchial hyperresponsiveness; NSAID, non steroidal anti-inflamatory drugs; OIT, oral immunotherapy; EMS, emergency medical services.

*Allergy skin prick tests and/or allergen-specific IgE blood tests do not predict reaction severity,* and anaphylaxis can occur in patients with high, low and even negative tests. A recent European Consensus concluded that it is very difficult if not impossible to accurately predict who is at risk of severe anaphylaxis: a number of risk factors acting together are involved (figure 3).^9^

Clearly someone with previous anaphylaxis is at risk of subsequent anaphylaxis. However, most children who present with anaphylaxis as their initial reaction do not experience further anaphylaxis. Ewan and Clark followed up 747 allergic children, of whom 220 had initial anaphylaxis to peanut/tree nuts; 25% had further accidental reactions over a median 3-year follow-up, with only one experiencing further anaphylaxis.^33^ Other studies report a higher rate of anaphylaxis in those with initial mild reactions. In a UK survey of 969 young people attending allergy clinics, 48% had experienced an accidental reaction in the previous year, with 245 (25%) having anaphylaxis.^6^ However, the occurrence of anaphylaxis is likely to depend on a number of factors, including dose or level of exposure^34^ (figure 3). In a unique study of 89 children with suspected peanut allergy, Wainstein *et al* demonstrated that up to 75% will have anaphylaxis if exposed to sufficient peanut at challenge.^35^ Thus, lack of prior anaphylaxis is more likely due to insufficient exposure rather than some inherent lack of predisposition. Importantly, *there are no data indicating that allergic reactions get worse with each subsequent exposure.* Nor is there any evidence to suggest that anaphylaxis risk ‘runs in the family’.

Various risk factors for severe anaphylaxis have been proposed, based on limited case series of fatal anaphylaxis. Interestingly, food-induced anaphylaxis is most common in the 0–5 age group, but death from anaphylaxis in this age group is rare.^2^ Teenagers and young adults appear to have an age-dependent predisposition towards severe outcomes, which cannot be easily explained by risk-taking behaviours.^2^ Asthma is considered a risk factor; however, in the UK Fatal Anaphylaxis Registry, 22% of cases did not have a prior diagnosis of asthma.^2^ Around 50% of children with food allergies have asthma: the vast majority will never have a severe allergic reaction, thus asthma has poor predictive value for severe reactions (although this does not negate the imperative to improve asthma control in food-allergic individuals as a means of reducing risk).^9^

Delays in treating with epinephrine are a risk factor for fatal outcome^10 36^: it is this, as well as our inability to predict severe reactions, which drives the provision of epinephrine autoinjectors. A summary of recent guidelines on who should be prescribed autoinjectors is summarised in table 1. Healthcare professionals must consider the patient/family preference: if prescription boosts patient confidence and allows them to lead a less restrictive life, then autoinjectors should be part of the management plan. However, this requires actual carriage: the autoinjectors need to be available at all times, otherwise prescription is pointless.

**Table 1:** Factors to be considered as part of the risk assessment on whether to prescribe epinephrine autoinjectors

	UK (BSACI)^48^	Europe (EAACI)^10^	Australia (ASCIA)^17^	Evidence
Previous history	Anaphylaxis and at risk of ongoing exposure Mild reaction to ‘trace’ amount of allergen History of cofactors (eg, exercise) impacting on reaction severity	**Anaphylaxis** Mild reaction to ‘trace’ amount of allergen **Venom allergy in adults with systemic symptoms**	**Anaphylaxis and at ongoing risk of exposure** Generalised urticaria alone without anaphylaxis due to insect sting in adults	Previous anaphylaxis indicates potential for future reactions, although risk of fatal anaphylaxis remains low.^7 15^ No evidence that individuals who react to very low amounts of allergen are more likely to experience severe anaphylaxis.^9^ Children with local or generalised skin rashes only to venom are at very low risk of anaphylaxis with subsequent stings.^10 48^
Allergen-specific risk factors	High-risk allergens, for example, nuts Allergen difficult to avoid	High-risk allergens, for example, nuts	High-risk allergens, for example, nuts, seafood	In the UK, cow’s milk and peanut/tree nuts are the most common cause of fatal anaphylaxis.^2^
Patient-specific risk factors	Teenage/young adults Food allergy* to high-risk allergens (eg, nuts) *and* other risk factors (eg, asthma) Raised baseline serum tryptase Limited access to emergency medical care, for example, remote location, social factors	Teenager or young adult with a food allergy* Food allergy* **and coexisting unstable or moderate–severe, persistent asthma** **Underlying mast cell disorders or raised baseline serum tryptase** Remote from medical help	Teenagers and young adults with food allergy Food allergy*and coexisting unstable or moderate–severe, persistent asthma **Underlying mast cell disorders (eg, systemic mastocytosis or raised baseline serum tryptase)** Limited access to emergency medical care, for example, remote location, foreign travel Cardiovascular disease	Data suggests a specific vulnerability to severe outcomes from food-induced allergic reactions in teenagers and young adults.^2 9^ Poor asthma control increases risk of severe reactions; most cases of fatal food-induced anaphylaxis have asthma, but asthma itself is poorly predictive of severe outcomes as it is so prevalent in food-allergic individuals.^9^ Underlying mast cell disorders are a known risk factor for venom and idiopathic anaphylaxis.^10 17 48^ Remote access to medical support causes delays in emergency treatment.

Factors in bold are specified as ‘absolute’ (EAACI) or ‘recommended’ (ASCIA) indications.

*Excluding pollen food allergy syndrome.

ASCIA, Australasian Society of Clinical Immunology and Allergy; BSACI, British Society for Allergy and Clinical Immunology; EAACI, European Academy of Allergy and Clinical Immunology.

Controversy exists over the number of autoinjectors to be prescribed. The BSACI and ASCIA in general recommend one device (for school children, one device for home and a second for school, while in the USA, physicians will generally prescribe two devices).^10 17^ In 2014, following an extensive review of epinephrine autoinjectors prompted by a coronial inquest, the Medicines and Healthcare products Regulatory Agency (MHRA) issued guidance that individuals at risk of anaphylaxis should carry two epinephrine autoinjectors at all times due to ‘uncertainties about the site of drug delivery and the speed of epinephrine action within the body’, which, together with device misuse or malfunction, might result in a second dose being needed.^37^ The BSACI guidance (issued after the 2014 statement) recommends a single device on the basis that one dose is usually effective for most reactions. The MHRA recently reiterated its policy,^38^ in line with new Department of Health guidance for school children at risk of anaphylaxis^39^ The MHRA review also addressed a concern that in some individuals (predominantly adolescent and adult women), the needle length in some autoinjectors may be insufficient to deliver an intramuscular (rather than subcutaneous) injection, although data to inform this are limited. At the current time, prescribing practice remains divided among UK healthcare professionals.

### Myth 8: ‘Epinephrine autoinjectors are overprescribed and overused in anaphylaxis’

Autoinjectors are underused to treat anaphylaxis in the community. In a study of infants aged 3–15 months with anaphylaxis (US definition), epinephrine was administered in under one-third, most commonly because the caregiver did not recognise the severity of reaction or the autoinjector was not available.^40^ In a UK study, only 16.7% of young people used an autoinjector to treat anaphylaxis, the most common reason being they did not recognise that the reaction needed treatment with epinephrine.^6^ A Scottish study among adolescents with previous anaphylaxis reported a number of barriers to the effective use of autoinjectors, including failure to recognise anaphylaxis, uncertainty and fear over how and when to use the autoinjector, and lack of carriage due to size/design.^41^ In the USA, these issues have led to some management plans (by FARE) offering the suggestion to use an epinephrine autoinjector for all reactions *regardless of severity*, but this remains controversial and is not accepted as standard practice among many healthcare professionals.^42^ It must be noted that anaphylaxis morbidity/mortality is no lower in the USA compared with UK and Australia where epinephrine is only recommended for reactions with respiratory or cardiovascular involvement^36^

### Myth 9: ‘Prescription of an epinephrine autoinjector in isolation is life-saving’

Optimal management of food-allergic patients and treatment of anaphylaxis has many facets and is not limited to a prescription for an epinephrine autoinjector. Improving patient/carer knowledge on the recognition and treatment of anaphylaxis, and addressing the complex psychosocial dimensions of allergic emergencies, form the cornerstone of successful anaphylaxis management.^6 41^ One-third of fatalities in the UK occur despite timely epinephrine administration.^20^ Epinephrine autoinjectors potentially buy valuable minutes while an emergency medical response is summonsed. Such devices need to be prescribed as part of a comprehensive management plan, which includes advice on dietary avoidance and on when to administer epinephrine. *Patients and their families need to be told to use their autoinjector in the event of any respiratory symptoms, where anaphylaxis might the cause,* irrespective of severity. Patients with asthma may not realise the importance of this; they may perceive mild wheezing following food allergen exposure as equivalent to their routine symptoms. Patients and their families ‘need to be provided with more constructive strategies and support’ than merely being told to ‘use your pen’.^42^

The BSACI management plans are available for download online (http://www.bsaci.org/about/pag-allergy-action-plans-for-children) and were recently updated to take into account changes in UK-wide legislation allowing the use of ‘spare’ epinephrine autoinjectors in schools. The BSACI plans, correctly completed, meet the requirements of the legislation and UK healthcare professionals are encouraged to use these plans where possible. Further information is available online (www.sparepensinschools.uk).

Correct positioning of the patient is important in anaphylaxis,^8 25^ something highlighted in MHRA guidance. Case series have highlighted the potential for a change in posture (eg, from sitting or lying to standing) to trigger decompensation and fatal event in some patients. Lying the patient supine with the lower limbs elevated will increase venous return and cardiac output. Patients with respiratory symptoms can be allowed to sit if this improves comfort, with their lower limbs elevated where possible. Sudden standing must be avoided, and patients with anaphylaxis must not be instructed to walk to a first aid room to use their autoinjector, as this may increase the risk of death.^8 25 36^

### Myth 10: ‘MMR and influenza vaccination are contraindicated in patients with previous anaphylaxis to egg’

A common misconception is that the influenza and measles, mumps, rubella (MMR) vaccines cannot be given to egg-allergic children, in particular those with previous anaphylaxis. The MMR vaccine is grown in chick fibroblast cell lines and does not contain detectable egg protein. Egg allergy, however severe, is not a contraindication.^43^ Influenza vaccines are prepared from viruses grown in embryonated hen’s eggs and can contain very low levels of ovalbumin. However, recent data have confirmed that both injected and intranasal forms of the vaccine are safe in egg-allergic children, including those with previous anaphylaxis.^44 45^ The ‘Green Book’^46^ and US guidelines^44^ now advise that these vaccines can be administered in primary care (or, in the case of the intranasal vaccine, schools), with the usual precautions taken for any vaccination. The only exception is those with previous life-threatening reactions to egg requiring intensive care, in whom there is little safety data (such reactions to egg are vanishingly rare); in any event, these patients (and their carers) may be better reassured if the vaccine is administered in hospital.

In contrast, yellow fever vaccine does contain small amounts of egg protein and has been reported to trigger anaphylaxis in some egg-allergic individuals. Desensitisation protocols for use in specialist centres are available,^47^ but administration is complicated by the need for such centres to be authorised to provide WHO certification. Currently, the authors are aware of only one UK paediatric centre (Evelina Hospital, London) where WHO certification can be issued following successful administration.

## Conclusions

Anaphylaxis is a severe, potentially life-threatening systemic allergic reaction, which constitutes a clinical emergency. Common misconceptions regarding anaphylaxis are summarised in table 2. Prompt assessment and management are essential, as delays in treatment are associated with fatal outcomes. Anaphylaxis is primarily a clinical diagnosis: patients/carers and health professionals must be appropriately trained to recognise and institute appropriate treatment with intramuscular epinephrine, as part of a comprehensive management plan. Epinephrine is the first-line treatment for anaphylaxis, but is underused. Changes in posture have been documented as a trigger for decompensation and fatal anaphylaxis. New management plans incorporating this advice, and which allow the use of ‘spare’ autoinjectors in schools, are available from the BSACI and via www.sparepensinschools.uk website.

**Table 2:** Common misconceptions in anaphylaxis and what current evidence reveals

Common ‘myths’	What evidence tells us
Myth 1: Anaphylaxis often results in death	Anaphylaxis can be life-threatening, but the majority of reactions do not result in severe outcomes
Myth 2: There are no hives so it can’t be anaphylaxis	Cutaneous symptoms (most commonly urticaria or ‘hives’) are absent in around 10% of anaphylaxis reactions
Myth 3: No trigger for the reaction is identified, therefore it is not anaphylaxis	In around 20% of cases, no trigger is identified; this is known as idiopathic anaphylaxis
Myth 4: Epinephrine is dangerous	Epinephrine given by intramuscular injection into the outer mid-thigh is very safe
Myth 5: Antihistamines can be used to treat anaphylaxis initially; epinephrine is only needed if symptoms worsen	Epinephrine, not antihistamines, is the first-line treatment for anaphylaxis
Myth 6: Corticosteroids prevent delayed or biphasic reactions in anaphylaxis	There is insufficient evidence to support the use of corticosteroids prevent delayed or biphasic reactions in anaphylaxis
Myth 7: Only children who have had anaphylaxis need an epinephrine autoinjector	It is very difficult—if not impossible—to accurately predict who is at risk of severe anaphylaxis
Myth 8: Epinephrine autoinjectors are overprescribed and overused in anaphylaxis	Autoinjectors are underused to treat anaphylaxis in the community
Myth 9: Prescription of an epinephrine autoinjector in isolation is life-saving	Optimal management of food allergic patients and treatment of anaphylaxis has many facets and is not limited to a prescription for an epinephrine autoinjector
Myth 10: MMR and influenza vaccination are contraindicated in patients with previous anaphylaxis to egg	Both vaccines are safe to administer in egg-allergic children, including those with previous anaphylaxis

## References

[1] Sampson HA, Muñoz-Furlong A, Campbell RL, et al Second symposium on the definition and management of anaphylaxis: summary report—Second National Institute of Allergy and Infectious Disease/Food Allergy and Anaphylaxis Network symposium. J Allergy Clin Immunol 2006;117:391–7. 10.1016/j.jaci.2005.12.1303 16461139

[2] Turner PJ, Gowland MH, Sharma V, et al Increase in anaphylaxis-related hospitalizations but no increase in fatalities: an analysis of United Kingdom national anaphylaxis data, 1992–2012. J Allergy Clin Immunol 2015;135:956–63. 10.1016/j.jaci.2014.10.021 25468198 PMC4382330

[3] Lee S, Hess EP, Lohse C, et al Trends, characteristics, and incidence of anaphylaxis in 2001–2010: a population-based study. J Allergy Clin Immunol 2017;139 10.1016/j.jaci.2016.04.029 PMC5182191 27378753

[4] Rudders SA, Arias SA, Camargo CA Trends in hospitalizations for food-induced anaphylaxis in US children, 2000–2009. J Allergy Clin Immunol 2014;134:960–2. 10.1016/j.jaci.2014.06.018 25109801

[5] Lieberman P, Camargo CA, Bohlke K, et al Epidemiology of anaphylaxis: findings of the American College of Allergy, Asthma and Immunology Epidemiology of Anaphylaxis Working Group. Ann Allergy Asthma Immunol 2006;97:596–602. 10.1016/S1081-1206(10)61086-1 17165265

[6] Noimark L, Wales J, Du Toit G, et al The use of adrenaline autoinjectors by children and teenagers. Clin Exp Allergy 2012;42:284–92. 10.1111/j.1365-2222.2011.03912.x 22181034

[7] Umasunthar T, Leonardi-Bee J, Hodes M, et al Incidence of fatal food anaphylaxis in people with food allergy: a systematic review and meta-analysis. Clin Exp Allergy 2013;43:1333–41. 10.1111/cea.12211 24118190 PMC4165304

[8] Muraro A, Roberts G, Worm M, et al Anaphylaxis: guidelines from the European Academy of Allergy and Clinical Immunology. Allergy 2014;69:1026–45. 10.1111/all.12437 24909803

[9] Turner PJ, Baumert JL, Beyer K, et al Can we identify patients at risk of life-threatening allergic reactions to food? Allergy 2016;71:1241–55. 10.1111/all.12924 27138061

[10] Muraro A, Halken S, Arshad SH, et al Primary prevention of food allergy. Allergy Eur J Allergy Clin Immunol 2014.

[11] Ma L, Danoff TM, Borish L Case fatality and population mortality associated with anaphylaxis in the United States. J Allergy Clin Immunol 2014;133:1075–83. 10.1016/j.jaci.2013.10.029 24332862 PMC3972293

[12] Jerschow E, Lin RY, Scaperotti MM, et al Fatal anaphylaxis in the United States, 1999–2010: temporal patterns and demographic associations. J Allergy Clin Immunol 2014;134:1318–28. 10.1016/j.jaci.2014.08.018 25280385 PMC4260987

[13] Mullins RJ, Wainstein BK, Barnes EH, et al Increases in anaphylaxis fatalities in Australia from 1997 to 2013. Clin Exp Allergy 2016;46:1099–110. 10.1111/cea.12748 27144664

[14] Motosue MS, Bellolio MF, Van Houten HK, et al Outcomes of emergency department anaphylaxis Visits from 2005 to 2014. J Allergy Clin Immunol Pract 2018;6 10.1016/j.jaip.2017.07.041 28941671

[15] Umasunthar T, Leonardi-Bee J, Turner PJ, et al Incidence of food anaphylaxis in people with food allergy: a systematic review and meta-analysis. Clin Exp Allergy 2015;45:1621–36. 10.1111/cea.12477 25495886

[16] Hu W, Grbich C, Kemp A When doctors disagree: a qualitative study of doctors' and parents' views on the risks of childhood food allergy. Health Expect 2008;11:208–19. 10.1111/j.1369-7625.2008.00506.x 18816318 PMC5060450

[17] ASCIA. Acute management of anaphylaxis. 2017 www.allergy.org.au/health-professionals/papers/acute-management-of-anaphylaxis-guidelines

[18] Rainbow J, Browne GJ Fatal asthma or anaphylaxis? Emerg Med J 2002;19:415–7. 10.1136/emj.19.5.415 12204988 PMC1725974

[19] Siddiqui S, Gonem S, Wardlaw AJ Advances in the management of severe asthma. Semin Respir Crit Care Med 2014;33:666–84. 10.1055/s-0032-1326964 23047316

[20] Pumphrey R Anaphylaxis: can we tell who is at risk of a fatal reaction? Curr Opin Allergy Clin Immunol 2004;4:285–90. 10.1097/01.all.0000136762.89313.0b 15238794

[21] Vetander M, Helander D, Flodström C, et al Anaphylaxis and reactions to foods in children—a population-based case study of emergency department visits. Clin Exp Allergy 2012;42:568–77. 10.1111/j.1365-2222.2011.03954.x 22417215

[22] Sampson HA, Mendelson L, Rosen JP Fatal and near-fatal anaphylactic reactions to food in children and adolescents. N Engl J Med 1992;327:380–4. 10.1056/NEJM199208063270603 1294076

[23] Grabenhenrich LB, Dölle S, Moneret-Vautrin A, et al Anaphylaxis in children and adolescents: The European Anaphylaxis Registry. J Allergy Clin Immunol 2016;137:1128–37. 10.1016/j.jaci.2015.11.015 26806049

[24] Soar J, Pumphrey R, Cant A, et al Emergency treatment of anaphylactic reactions—guidelines for healthcare providers. Resuscitation 2008;77:157–69. 10.1016/j.resuscitation.2008.02.001 18358585

[25] Simons FE, Ardusso LR, Bilò MB, et al 2012 update: World Allergy Organization guidelines for the assessment and management of anaphylaxis. Curr Opin Allergy Clin Immunol 2012;12:389–99. 10.1097/ACI.0b013e328355b7e4 22744267

[26] Francis A, Fatovich DM, Arendts G, et al Serum mast cell tryptase measurements: sensitivity and specificity for a diagnosis of anaphylaxis in emergency department patients with shock or hypoxaemia. Emerg Med Australas 2017 10.1111/1742-6723.12875 29094472

[27] De Schryver S, Halbrich M, Clarke A, et al Tryptase levels in children presenting with anaphylaxis: temporal trends and associated factors. J Allergy Clin Immunol 2016;137:1138–42. 10.1016/j.jaci.2015.09.001 26478007

[28] Tejedor Alonso MA, Moro Moro M, Múgica García MV, et al Incidence of anaphylaxis in the city of Alcorcon (Spain): a population-based study. Clin Exp Allergy 2012;42:578–89. 10.1111/j.1365-2222.2011.03930.x 22417216

[29] Gorska L, Chelminska M, Kuziemski K, et al Analysis of safety, risk factors and pretreatment methods during rush hymenoptera venom immunotherapy. Int Arch Allergy Immunol 2008;147:241–5. 10.1159/000142048 18594155

[30] Alqurashi W, Ellis AK Do corticosteroids prevent biphasic anaphylaxis? J Allergy Clin Immunol Pract 2017;5:1194–205. 10.1016/j.jaip.2017.05.022 28888249

[31] Lee L, Bellolio MF, Hess EP, et al 34 time of onset and predictors of biphasic anaphylactic reactions: a systematic review and meta-analysis of the literature. Ann Emerg Med 2014;64:S13 10.1016/j.annemergmed.2014.07.059 25680923

[32] NICE. Anaphylaxis Guideline. www.nice.org.uk/guidance/CG134

[33] Ewan PW, Clark AT Efficacy of a management plan based on severity assessment in longitudinal and case-controlled studies of 747 children with nut allergy: proposal for good practice. Clin Exp Allergy 2005;35:751–6. 10.1111/j.1365-2222.2005.02266.x 15969666

[34] Dubois AEJ, Turner PJ, Hourihane J, et al How does dose impact on the severity of food-induced allergic reactions, and can this improve risk assessment for allergenic foods?: report from an ILSI Europe Food Allergy Task Force Expert Group and Workshop. Allergy 2018 10.1111/all.13405 PMC6032860 29331070

[35] Wainstein BK, Studdert J, Ziegler M, et al Prediction of anaphylaxis during peanut food challenge: usefulness of the peanut skin prick test (SPT) and specific IgE level. Pediatr Allergy Immunol 2010;21(4 Pt 1):603–11. 10.1111/j.1399-3038.2010.01063.x 20444154

[36] Turner PJ, Jerschow E, Umasunthar T, et al Fatal anaphylaxis: mortality rate and risk factors. J Allergy Clin Immunol Pract 2017;5:1169–78. 10.1016/j.jaip.2017.06.031 28888247 PMC5589409

[37] MHRA. Adrenaline auto-injectors : a review of clinical and quality considerations. Med Healthc Prod Regul Agency 2014.

[38] Gov.UK. Adrenaline auto-injectors: updated advice after European review. https://www.gov.uk/drug-safety-update/adrenaline-auto-injectors-updated-advice-after-european-review

[39] Gov.UK. Using emergency adrenaline auto-injectors in schools. https://www.gov.uk/government/publications/using-emergency-adrenaline-auto-injectors-in-schools

[40] Fleischer DM, Perry TT, Atkins D, et al Allergic reactions to foods in preschool-aged children in a prospective observational food allergy study. Pediatrics 2012;130:e25–e32. 10.1542/peds.2011-1762 22732173 PMC3382915

[41] Gallagher M, Worth A, Cunningham-Burley S, et al Epinephrine auto-injector use in adolescents at risk of anaphylaxis: a qualitative study in Scotland, UK. Clin Exp Allergy 2011;41:869–77. 10.1111/j.1365-2222.2011.03743.x 21481022

[42] Turner PJ, DunnGalvin A, Hourihane JO The Emperor has no symptoms: the risks of a blanket approach to using epinephrine autoinjectors for all allergic reactions. J Allergy Clin Immunol Pract 2016;4:1143–6. 10.1016/j.jaip.2016.05.005 27283056 PMC5123619

[43] UK Medicines Information. Update to Chapter 6 of the green book—egg allergy (41–48). 2017 http://www.medicinesresources.nhs.uk/en/Medicines-Awareness/Guidance-and-Advice/Drug-Prescribing/Update-to-Chapter-6-of-the-green-book--egg-allergy/

[44] Greenhawt M, Turner PJ, Kelso JM Administration of influenza vaccines to egg allergic recipients: a practice parameter update 2017. Ann Allergy Asthma Immunol 2018;120:49–52. 10.1016/j.anai.2017.10.020 29273128

[45] Turner PJ, Southern J, Andrews NJ, et al Safety of live attenuated influenza vaccine in atopic children with egg allergy. J Allergy Clin Immunol 2015;136:376–81. 10.1016/j.jaci.2014.12.1925 25684279 PMC4534767

[46] Gov.UK. Chapter 19: Influenza. https://www.gov.uk/government/uploads/system/uploads/attachment_data/file/663694/Greenbook_chapter_19_Influenza_.pdf

[47] Rutkowski K, Ewan PW, Nasser SM Administration of yellow fever vaccine in patients with egg allergy. Int Arch Allergy Immunol 2013;161:274–8. 10.1159/000346350 23548550

[48] Ewan P, Brathwaite N, Leech S, et al BSACI guideline: prescribing an adrenaline auto-injector. Clinical & Experimental Allergy 2016;46:1258–80. 10.1111/cea.12788 27893945

